# TC-PTP regulates the IL-7 transcriptional response during murine early T cell development

**DOI:** 10.1038/s41598-017-13673-w

**Published:** 2017-10-16

**Authors:** K. A. Pike, T. Hatzihristidis, S. Bussières-Marmen, F. Robert, N. Desai, D. Miranda-Saavedra, J. Pelletier, M. L. Tremblay

**Affiliations:** 1 Rosalind and Morris Goodman Cancer Centre, McGill University, Montréal, QC H3A 1A3 Canada; 2 Division of Experimental Medicine, Department of Medicine, McGill University, Montréal, QC H3A 1A3 Canada; 3 Department of Biochemistry, McGill University, Montréal, QC H3A 1A3 Canada; 4 Centro de Biología Molecular Severo Ochoa, CSIC/Universidad Autónoma de Madrid, 28049 Madrid, Spain; 5 Department of Computer Science, University of Oxford, Wolfson Building Parks Road, OXFORD, OX1 3QD UK

## Abstract

Cytokines play a critical role in directing the discrete and gradual transcriptional changes that define T cell development. The interleukin-7 receptor (IL-7R), via its activation of the JAK-STAT pathway, promotes gene programs that change dynamically as cells progress through T cell differentiation. The molecular mechanism(s) directing differential gene expression downstream of the IL-7R are not fully elucidated. Here, we have identified T cell protein tyrosine phosphatase (TC-PTP), also known as PTPN2, as a negative regulator of IL-7R-STAT signaling in T cell progenitors, contributing to both the quantitative and qualitative nature of STAT-gene targeting. Novel genetic strategies used to modulate TC-PTP expression demonstrate that depletion of TC-PTP expression heightens the phosphorylation of STAT family members, causing aberrant expression of an interferon-response gene profile. Such molecular re-programming results in deregulation of early development checkpoints culminating in inefficient differentiation of CD4^+^CD8^+^ double positive cells. TC-PTP is therefore shown to be required to safeguard the dynamic transcriptome necessary for efficient T cell differentiation.

## Introduction

The transcriptional response elicited by interleukin-7 (IL-7) plays a critical role during T cell development. While recent thymic emigrants lack IL-7 receptor (IL-7R) expression, it is upregulated on early double negative (DN) thymocytes^1^. The DN thymic population is identified by the lack of expression of the T cell receptor (TCR) and the co-receptors CD4 and CD8, and is further subdivided based on CD44 and CD25 expression^2^. The IL-7R can first be detected on the surface of DN2 cells and high levels of expression are maintained on DN3 cells^1^. During a period of quiescence, the recombinase genes (*Rag1* and *Rag2*) are induced and the TCR gene segments are rearranged^3,4^. Following productive rearrangement of the β-chain, the TCRβ protein is expressed in association with the pre-Tα protein, forming the pre-TCR^5,6^. Integrated signaling downstream of the pre-TCR, Notch and IL-7R enforce β-selection leading to the down-modulation of RAG1 and RAG2, and the increased expression of the trophic factors CD71 and CD98^7^. Following a robust period of proliferation, β-selected DN3 cells differentiate into cycling DN4 cells and gradually downregulate IL-7R expression^1^.

The IL-7R mediates its effects on gene expression through the Janus kinases (JAK)-signal transducer and activator of transcription (STAT) signaling pathway, STAT5 being the dominant STAT family member activated downstream of the IL-7R. The nature of genes targeted by IL-7-STAT5 changes dynamically as T cell progenitors transition through early T cell differentiation^8^. Initial characterization of IL-7 deficient mice identified IL-7 as a survival factor for DN2 and DN3 cells. As evidence, the reduction in the absolute number of DN2 and DN3 cells observed in IL-7 and IL-7 receptor (IL-7R) deficient mice, can be partially rescued by transgenic expression of human BCL-2^9,10^ or depletion of pro-apoptotic Bcl-2 family members^11,12^. Indeed DN2, DN3 and DN4 cells have increased levels of STAT5 phosphorylation and Bcl-2 expression in response to IL-7, correlating with an increase in cell viability^8,13^. Following β-selection however, IL-7R signaling also promotes the expression of genes involved in cell metabolism, cell proliferation and the repression of premature differentiation^8^.

Although IL-7R signaling is required to promote progenitor survival, direct T lineage commitment and ensure that metabolic demands are met, over-expression of IL-7 can be deleterious to T cell lymphopoiesis. A comparison of IL-7 transgenic mouse strains, demonstrated that expression of high levels of thymic IL-7 reduces T cell proliferation and blocks CD4^+^CD8^+^ DP production^14^. Mechanistically, heightened IL-7R signaling was shown to increase the expression of the inhibitory molecule SOCS3, causing a hypo-activation of the PI3K-Akt pathway and Notch-1 signaling. As a result, commitment to the T cell lineage is hindered and B cell differentiation is promoted in the thymus^15^.

The mechanisms maintaining the threshold of IL-7R signaling and regulating the specificity of IL-7R-STAT5 gene targeting in progenitors as they differentiate is unclear, and speaks to the larger question of cytokine receptor (CytR) specificity in the field of cytokine biology. T cell protein tyrosine phosphatase (TC-PTP) is a non-receptor PTP that negatively regulates JAK-STAT signaling in response to growth factor and cytokine stimulation. TC-PTP deficient (*tc-ptp* ^−/−^) mice exhibit thymic atrophy and succumb to anemia and progressive systemic inflammatory disease between 3 and 5 weeks of age^16^. TC-PTP binds and dephosphorylates JAK1 and JAK3, and TC-PTP loss is associated with elevated activation of STAT1 and STAT5^17^ in macrophages, B cells and T cells. Similarly, *tc-ptp* ^−/−^ thymocytes display heightened STAT1 phosphorylation in response to IFNγ or IFNα stimulation^17^. While it has been suggested that TC-PTP depletion results in impaired survival and/or differentiation of ETP and DN3 subsets, it has remained unclear whether these effects are cell intrinsic or due to the systemic inflammation and elevated IFNγ levels observed in *tc-ptp* ^−/−^ mice.

Mice harboring a T cell specific-TC-PTP deficiency have increased susceptibility to inflammation and autoimmunity due to heightened antigen driven T cell activation. Mechanistically it was demonstrated that TC-PTP inactivates Src family kinases downstream of the TCR, thereby contributing to the threshold of TCR activation. Similarly, elevated TCR signaling in TC-PTP deficient CD4^+^CD8^+^ DP thymocytes was shown to cause increased positive selection, resulting in a decreased ratio of SP/DP thymocytes, despite no change in the number of DPs. The genetic strategy used to generate the T cell specific TC-PTP knock-out however, leads to complete gene excision only at the DP stage of T cell development^18^. As such, the role of TC-PTP in DN cells could not be addressed.

To examine the intrinsic role of TC-PTP in DN cells, we used unique *in vitro* differentiation assays to monitor progression through each developmental checkpoint. We have identified TC-PTP as a regulator of STAT activation in early T cell progenitors. TC-PTP depletion resulted in heightened STAT5 and STAT1 phosphorylation, expression of an IFN gene signature and the impairment of differentiation beyond the β-selection checkpoint. We conclude that TC-PTP is required to promote proper gene expression in response to IL-7, impacting both the quantity and quality of the IL-7 mediated transcriptional profile.

## Materials and Methods

### Mice

*Ptpn2* ^−/−^ (TC-PTP^−/−^) mice have been previously described in our laboratory^16^. Heterozygous *Ptpn2* ^+/−^ mice were bred to generate littermate controls for each experiment. *Rag2* ^−/− ^^4^ mice were generously provided by the laboratory of Dr. R. Jones (McGill University, Montreal, Canada).

### Stem cell isolation

*(Fetal-liver)* Fetal liver cells were harvested at day 14–15 of gestation and single-cell suspensions were generated and filtered through-40 micron filters. Antibody complement lysis was performed to enrich CD24^−/lo^ cells as previously described^19^. *(Bone marrow)* Bone marrow was isolated from femurs and tibias and cultured in the presence of 50 ng/ml IL-6, 20 ng/ml IL-3 and 50 ng/ml SCF (Peprotech) for 16 hours prior to FACS-sorting Lin^-^c-Kit^+^Sca-1^high^ cells.

### Generation of shRNA vectors

Seventeen shRNAs targeting TC-PTP were designed using the siDirect siRNA library (http://sidirect2.rnai.jp/) and the NM_008977.3 (Ensembl) sequence of TC-PTP. Modified 293T cells were transfected with the pMLP vector expressing the shRNA and a puromycin resistant gene under the PGK promoter, and GFP expression protein under IRES, as described in Premsrirut *et al*. Two days following the transfection, the viral supernatant produced was used to infect NIH3T3 cells for 48 hours before starting puromycin treatment (PUR.333 Bioshop, 5 ug/ul) for 5 days. GFP expression was assessed with EVOS microscope and the expression of TC-PTP by western blotting to verify the level of KD generated in NIH3T3 infected cells. To test the efficiency of the shRNAs in minimal conditions and mimic the infection of NIH3T3 cells with only a single-copy (SC) of each shRNA, a serial dilution of the viral supernatants was performed to obtain a multiplicity of infection of one. A shRNA against firefly luciferase was also used as a control throughout the shRNA efficiency testing.

### Generation of tetracycline inducible shRNA mouse model

Selected shRNA oligonucleotides were cloned into the pBS31 vector. Generated vectors were coelectroporated with pCAGs-Flpe into 2 × 10^7^ D34 ES cells (gifts from the McGill Transgenic Core facility and Dr. Pelletier, McGill University, respectively). Clones were selected by hygromycin treatment for 8 days (GIBCO, 8 μg/ml). To confirm the capacity to induce shRNA expression, D34 clones were infected with Cre recombinase viral supernatant produced from 293T cells transfected with Cre recombinase (2 μg/ul, gift from Dr. Arnim Pause, McGill University, Montreal, Canada), and treated with doxycycline hyclate (1μg/ml,D9891, Sigma) for 4 days.

Selected positive D34 clones expressing the chosen shRNA were injected into C57Bl/6 blastocysts (in collaboration with the McGill Transgenic Core facility). Chimeric mice were mated with C57BL/6 mice for 8 generations and their offspring mated with Meox2-cre (generous gift from the McGill Transgenic Core facility) to obtain the experimental mice (TSI/cre+). Genotyping of wildtype and mutant alleles was assessed by a series of PCRs: TRE-PCR for the presence of the shRNA on one or both alleles, rtTA-PCR and ROSA-PCR for the presence of rtTA on one or both alleles, and Cre-PCR for the expression of the cre-recombinase. Primers and amplification conditions are summarized in supplemental Material and Methods. The water treatment for experimental animals consists of doxycycline hyclate (1 mg/ml, D9891 Sigma) with dextrose (5 g/100 ml, D9434 Sigma) filtered with a 0.2 uM filter (V50 Sarstedt). The treatment lasted for 7 days unless otherwise indicated, changing the water every 2–3 days. Live GFP imaging on mice was performed using the IVIS 100 *in vivo* imaging system (PerkinElmer) and the luminescence is displayed as total luminous flux over time (photons/second). Animal protocols were in accordance with the regulations of the Canadian Council on Animal Care and approved by the McGill University animal care committee.

### RNA isolation, reverse transcriptase and real-time PCR

Total RNA was extracted from co-culture derived DN3s. RNA extraction was performed using the TRIzol® Reagent (Invitrogen) according to the manufacturer’s instructions. Any potential contaminating genomic DNA was degraded using the DNAse I RiboPure kit (Life Technologies). RNA was transcribed to cDNA using the SuperScript III Reverse Transcriptase Kit (Life Technologies) according to the manufacturer’s instructions and qRT-PCR was performed on a LightCycler 480 using SYBR Green master mix according to manufacturer’s instructions. RNA was quantified using the NanoDrop 100 Spectrophotometer (Thermo Scientific). Statistical analysis of gene expression obtained by qRT-PCR was performed using one-way ANOVA with a Tukey post-test.

### Flow cytometry and cell isolation

Surface antigens were detected using fluochrome conjugated antibodies, specific for CD25, CD44, CD4, CD8, CD24, CD71, and CD98 (eBiosciences). Intracellular proteins were detected by initially fixing and permeabilizing cells with BDCytofix/Cytoperm (BD Biosciences). Intracellular TCRβ and Ki67 were detected using fluochrome conjugated antibodies. All samples were collected on a FACSCalibur instrument (BD Biosciences) and analyzed with FlowJo software (Tree Star).

### Immunoblotting

Total cell lysates were generated using modified RIPA buffer (50 mM Tri-HCl, pH 7.5, 150 mM NaCl, 0.25% sodium deoxycholate, 1% NP-40). Lysates were resolved by SDS-PAGE and transferred for immunoblotting. The following antibodies used for western blotting were purchased from Cell Signaling Technology and used at 1:1000: rabbit anti-phospho-STAT3 (Y705), rabbit anti-STAT3 (79D7), rabbit anti-phospho-STAT1 (Y701) (58D6), rabbit anti-STAT1, rabbit anti-phospho-STAT5 (Y694) and rabbit anti-STAT5 (3H7). The mouse anti-calnexin antibody (1:5000) was kindly provided by Dr. Bergeron (McGill University, Montreal, Canada), while the rabbit anti-PTP1B (ABS40, 1:1000) antibody was obtained from Millipore. Mouse monoclonal anti-TC-PTP antibody was described previously. Secondary antibodies, goat anti-mouse Ig-HRP and goat anti-rabbit-Ig-HRP (Jackson Laboratories), were used at 1:5000.

### Extraction of Differentially Expressed Genes

A standard Tophat-Cufflinks pipeline was used to determine which genes were significantly differentially expressed in the RNA-sequencing datasets. The RNA reads were mapped to the UCSC mouse genome (GRCm38/mm10) using Tophat (version 2.0.12) with default parameters and no coverage search. Once alignments were completed, Cufflinks (version 2.1.1) was used with default parameters to reconstruct transcripts and quantify expression. Cuffdiff was then used to determine which genes were significantly differentially expressed between the knock-out and wild-type samples. The genes included in Table 1 have p-values of 5E-05 or less.

**Table 1:** List of restricted genes upregulated in *tc-ptp* ^−/−^ DN3 cells differentiated in OP9-DL1 co-culture.

	Gene Symbol	WT AVG FPKM	KO AVG FPKM	log2 (fold_change)
MHC I	H2-Q8	3.56484	272.398	6.25574
	H2-Q7	20.7694	614.774	4.88752
	H2-Q5	4.70419	70.6167	3.90799
	Nlrc5	14.4204	79.3806	2.46068
Slfn proteins	Slfn1	0.629762	49.7789	6.30458
	Slfn5	9.76887	74.2155	2.92546
IFN-inducible GTPases	Gbp2	14.2193	87.9553	2.62892
	Gbp5–001	6.25477	31.7183	2.34229
	Gbp5–201	6.08113	57.5039	3.24125
	Irgb10	21.965	129.957	2.56475
	Igtp	59.4418	289.992	2.28646
	Mx2	1.47454	31.8572	4.43328
Oas	Oas1a	13.9428	81.2341	2.54256
	Oas2	8.88515	75.7074	3.09097
	Oas3	9.68588	60.4577	2.64197
Pro-inflammatory caspase	Casp1	9.67395	52.0372	2.42737
	Casp4 (Caps 11)	8.1896	34.8142	2.08781
IFIT, IFITM and IFI proteins	Ifi44	6.74574	93.2798	3.78952
	Ifit3b	2.17103	28.969	3.73805
	Ifit3	12.7917	147.588	3.52829
	Ifitm1	45.5963	258.185	2.50142
	Ifi27l2a (Isg12)	106.989	557.24	2.38084
	Ifit1	15.4928	78.9642	2.3496
	GM14446 (Ifit1c)	9.47289	126.534	3.73958
Misc. interferon response genes	Gzmb	3.38683	46.1458	3.76819
	Gm4951 (Ifgg2a)	15.6324	166.136	3.40975
	Zbp1	29.7782	193.714	2.7016
	Samhd1	9.7597	60.879	2.64104
	Stat1	60.509	260.381	2.1054
	Spats2l	0.0778114	2.1568	4.79276
	Ly6a	64.8023	748.919	3.53069
	Rnf213	35.776	171.866	2.26422
	Lif	21.5133	62.1575	2.09417
	Cmpk2	5.59195	25.8325	2.20777
	Rsad2 (viperin)	2.15876	18.7161	3.11601
	Ctss (CathepsinS)	5.46336	32.7083	2.5818
	Ms4a4b (Ly116)	64.4905	280.406	2.12036
	Ube2l6	6.86829	55.3052	3.00939
	Usp18	30.253	149.024	2.3004
	Pydc3	12.6929	50.9631	2.00544
	Pydc4	3.11267	34.6213	3.47543
Non-interferon response genes	Ly6c1	23.0249	230.51	3.32356
	Gm12253	1.95832	13.2333	2.75649

Differentially expressed genes between *tc-ptp* ^+/+^ and *tc-ptp* ^−/−^ DN3 cells identified by at least a 2.0 log2 fold difference and a p-value of 5E-05 or less. The average fragments per kilobase of transcript per million mapped reads (FPKM) values from 3 biological replicates as detected by RNA-Seq are indicated.

### Retroviral Gene Transfer

Recombinant retrovirus was pseudotyped with VSV-g protein to enhance infectivity. pMLP vectors encoding either Luciferase or TC-PTP targeting shRNA were co-transfected with pCL-ECO and pCMV-VSV-G into 293T cells. Viral supernatants were collected at 48 h post-transfection. In a 24-well plate, 1 ml of viral supernatant was aliquoted per well to which 0.5 × 10^6^ (0.5 mL) CD24^−/lo^-depleted FL cells or total BM cells were added. FL infections were supplemented with 10 μg/mL of hexadimethrine bromide (Sigma-Aldrich), 5 ng/mL of mIL-7, 5 ng/mL of hFlt-3L, and 10 ng/mL of mSCF (Peprotech). BM infections were supplemented with 10 μg/mL of hexadimethrine bromide, 50 ng/ml IL-6, 20 ng/ ml IL-3 and 50 ng/ml SCF (Peprotech). Plates were then centrifuged at 1,000 × *g* for 1.5 hours at room temperature. Sixteen to 24 hours after transduction, Lin^-^c-Kit^+^Sca-1^high^ GFP^+^ cells were FACS sorted.

### *In Vitro* T-cell differentiation

Sorted hematopoietic progenitors, transduced or non-transduced, were plated at 4,000–6,000 cells per well on a 24-well plate containing a confluent layer of OP9-DL1 cells and differentiated as described previously^20^. Co-cultures were maintained in alpha MEM supplemented with 20% FCS, 5 μg/mL of hFlt-3L, and 5 μg/mL of mIL-7 (Peprotech) unless otherwise stated, and passaged onto fresh confluent OP9-DL1 every 4 days.

## Results

### TC-PTP deficiency impairs the early stages of thymopoiesis

**Figure 1:**
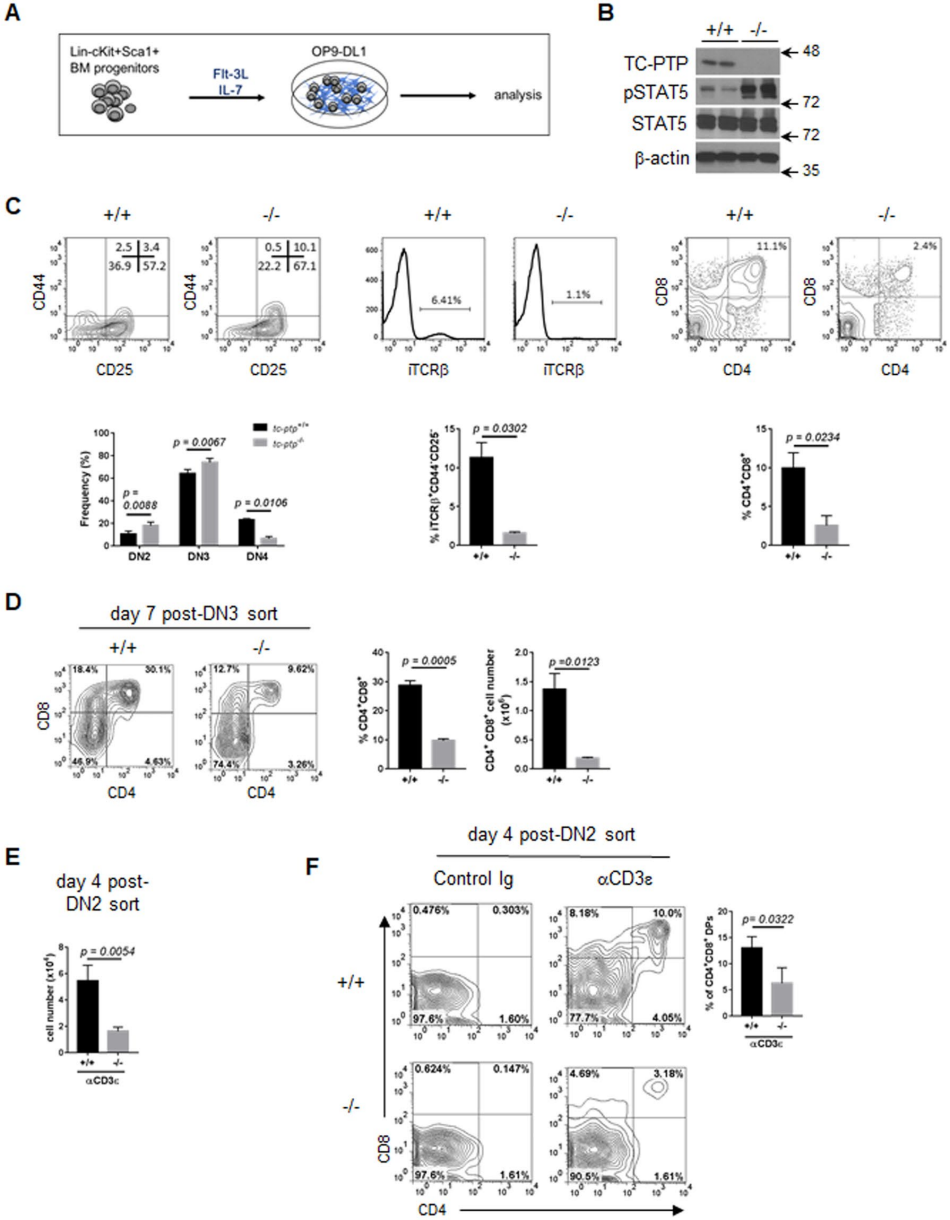
Deregulated T cell development caused by intrinsic progenitor defect. (**A**) Schematic of experimental protocol used to differentiate LSKs isolated from from *tc-ptp* ^+/+^ or *tc-ptp* ^−/−^ bone marrow in the OP9-DL1 co-culture system. (**B**) Differentiating progenitors were isolated from OP9-DL1 co-cultures on day 21 and total protein lysates resolved by SDS-PAGE. Full-length blots are presented in Supplementary Information. (**C**) On day 20 of co-culture, progenitors were isolated and the expression of differentiation markers determined by flow cytometry. Representative FACS plots are shown, while the mean+/− of SEM of three independent experiments are represented graphically. Numbers with FACS profiles indicate the frequency of gated populations. Gates were determined by FMO and isotype control staining. (**D**) Pre-β-selected DN3 cells were FACS-sorted based on size and cultured with OP9-DL1 cells for 7 days after which progenitors were isolated. Representative contour plots of 4 experiments are shown with numbers indicating the frequency of gated populations. The mean+/− SEM frequency and absolute number of CD4^+^CD8^+^ cells as determined by flow cytometry is shown. (**E**, **F**) FACS-sorted *tc-ptp* ^+/+^ and *tc-ptp* ^−/−^ DN2 cells were cultured on OP9-DL1 cells either in the presence or absence of anti-CD3ε cross-linking antibodies. The mean+/− SEM absolute cell number following 4 days of culture (**E**), representative contour plots of DP differentiation and the mean percentage of DPs (**F**) from three independent experiments are reported. P-values were determined by a two-tailed paired t-test (**C**,**D**) or two-tailed unpaired t-test (**F**).

T cell differentiation cultures were established by FACS-sorting Lin^−^c-Kit^+^Sca-1^HI^ (LSKs) cells from the bone marrow (BM) of *tc-ptp* ^+/+^ and *tc-ptp* ^−/−^ mice. Sorted cells were cultured with OP9-DL1 cells in the presence of Flt3L and IL-7 and T cell development was monitored by flow cytometry over time (Fig. 1A). Following 21 days of culture, differentiating progenitors were isolated and TC-PTP deficiency confirmed by western blotting. In addition, an elevation of STAT5 phosphorylation was observed, indicating deregulated JAK-STAT signaling in T cell progenitors (Fig. 1B).

By day 20, an accumulation of DN2 and DN3 cells was observed in *tc-ptp* ^−/−^ co-cultures compared to control cultures. A decrease in intracellular TCRβ^+^ (iTCRβ^+^) DN3 cells correlated with a reduction in the frequency of DN4 and DP cells (Fig. 1C).

To further investigate the apparent diminished efficiency of DP differentiation, pre-β-selected DN3 cells were FACS-sorted based on size from *tc-ptp* ^+/+^ and *tc-ptp* ^−/−^ thymii and cultured with OP9-DL1 cells. Following 7 days of culture, a reduction in the frequency and absolute number of DPs was observed in *tc-ptp* ^−/−^ co-cultures. While *tc-ptp* ^+/+^ co-cultures generated 1.27 ± 0.27 × 10^6^ DP cells whereas *tc-ptp* ^−/−^ produced only 0.18 ± 0.02 × 10^6^ DPs (Fig. 1D).

The reduced DN3-DP differentiation suggests a defect at the β-selection checkpoint. To by-pass β-selection, DN2 cells were sorted from *tc-ptp* ^+/+^ and *tc-ptp* ^−/−^ thymii and cultured with OP9-DL1 cells in the presence or absence of anti-CD3ε cross-linking antibodies^21–24^. Anti-CD3ε antibodies have been shown to induce CD3-mediated signaling that mimics pre-TCR signaling and drives transition through the β-selection checkpoint. Following 4 days of culture in the presence of the anti-CD3ε antibody, *tc-ptp* ^−/−^ cultures produced 3.3-fold fewer cells than control cultures (Fig. 1E). While, incubation with anti-CD3ε promoted DP cell differentiation in both *tc-ptp* ^+/+^ and *tc-ptp* ^−/−^ cultures, the frequency of DP cells was 2.1 fold reduced in t*c*-*ptp* ^−/−^ co-cultures compared to control *tc-ptp* ^+/+^ co-cultures (Fig. 1F). As such, it can be concluded that TC-PTP depletion impairs differentiation through a mechanism independent of pre-TCR signaling.

### TC-PTP depletion by shRNA recapitulates the tc-ptp^−/−^ T cell development defect

**Figure 2:**
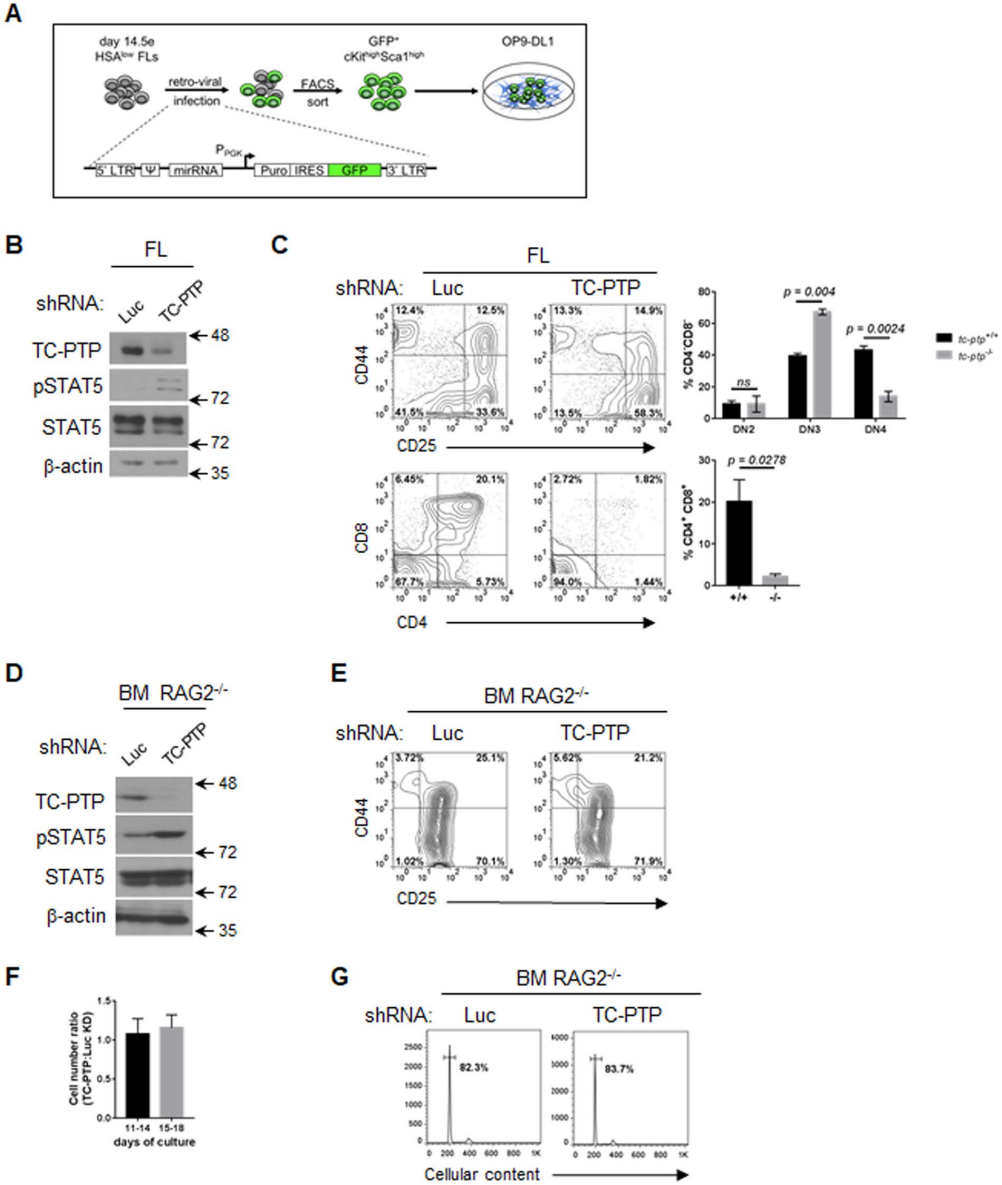
TC-PTP depletion by shRNA recapitulates intrinsic progenitor defect during T cell development. (**A**) Schematic representation of the experimental strategy to transduce TC-PTP targeting shRNA into FL-derived LSKs prior to differentiation in the OP9-DL1 co-culture system. (**B**) Following 12 days of culture, progenitors were isolated and protein lysates resolved and probed to confirm TC-PTP depletion. Full-length blots are presented in Supplementary Information. (**C**) Representative contour plots are shown, while the mean+/− SEM of each developmental subset in 3 independent experiments are show. (**D**) RAG2^−/−^ bone marrow was transduced with TC-PTP targeting shRNA after which transduced RAG2^−/−^ LSKs were differentiated in the co-culture system. Protein lysates were resolved and TC-PTP depletion confirmed by western blotting. Full-length blots are presented in Supplementary Information. (**E**) Representative FACS plots of T cell differentiation of transduced RAG2^−/−^ LSK following 12 days of cultures. (**F**) Mean+/− SEM of the ratio of the absolute cell number of Luc KD to TC-PTP KD co-cultures on day 14 and day 18, in 3 independent experiments. (**G**) Propidium iodide staining was performed for cell cycle analysis of transduced RAG2^−/−^ progenitors on day 16 of co-culture. Representative histograms of 3 independent experiments are shown. Numbers within FACS plots indicate the population frequency. Statistical significance was determined by a two-tailed paired t-test (**C**).

In a complimentary approach, TC-PTP expression was depleted from fetal-liver (FL) derived LSKs by the retroviral delivery of a short hairpin RNA (shRNA) targeting either TC-PTP (TC-PTP shRNA) or luciferase (Luc shRNA) as a control. Expression of GFP by an independent promoter permitted the purification of transduced cells. Fetal liver-derived enriched hematopoietic progenitors (HSA^low^) were transduced and GFP^+^ LSKs^+^ were FACS-sorted 24 hours later. Purified TC-PTP knock-down (KD) and Luc KD LSKs were then cultured with OP9-DL1 cells (Fig. 2A). TC-PTP depletion and increased STAT5 phosphorylation were confirmed by western blotting (Fig. 2B). Flow cytometric analysis of day 14 co-cultures indicated that depletion of TC-PTP resulted in delayed T cell differentiation, as evidenced by an increased frequency of DN3 cells and a reduced frequency of DPs (Fig. 2C).

### TC-PTP deficiency does not impair cellular expansion prior to β-selection

To determine if TC-PTP loss impairs the expansion of T cell progenitors prior to β-selection, T cell progenitors were differentiated from *Rag2* ^−/−^ bone marrow LSKs transduced with the TC-PTP targeting shRNA vector (Fig. 2D). A comparable distribution of DN2 and DN3 was observed between *Rag2* ^−/−^ TC-PTP KD and Luc KD co-cultures (Fig. 2E). In addition, the ratio of Luc KD and TC-PTP KD in three independent experiments was comparable, correlating with similar cell cycle progression (Fig. 2F,G).

### TC-PTP deficiency promotes the expression of an IFN-inducible gene profile

**Figure 3:**
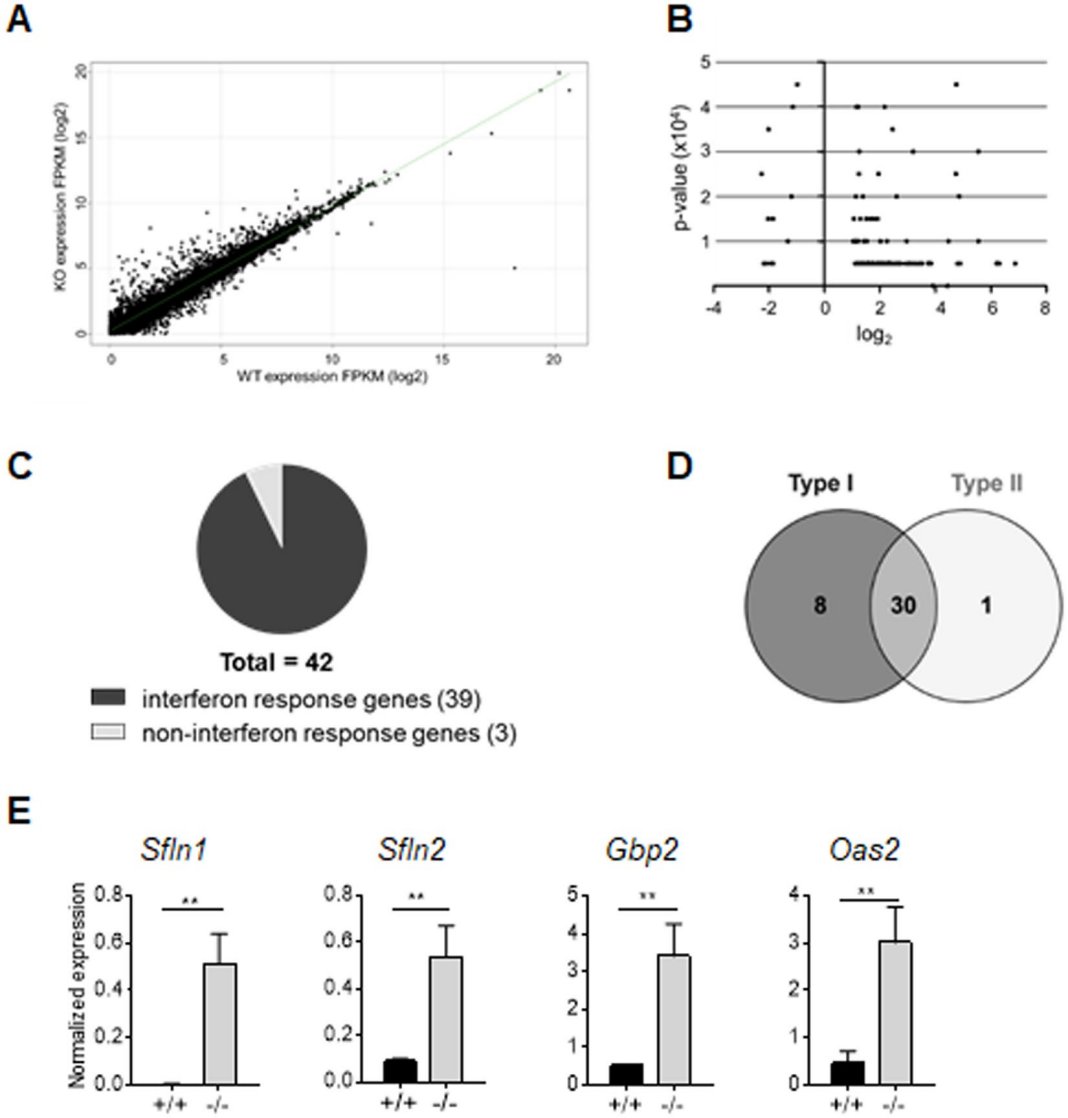
Induction of interferon response gene profile in the absence of TC-PTP. *Tc-ptp* ^+/+^ and *tc-ptp* ^−/−^ DN3 cells were differentiated on OP9-DL1 cells from LSKs. RNA from three independent cultures was extracted and sequenced. Standard Tophat-Cufflinks pipeline was used to align RNA-seq reads and study gene expression. (**A**) Scatter plot of log2 FPKM values for all UCSC mouse genome (GRCm38/mm10) genes for *tc-ptp* ^+/+^ versus *tc-ptp* ^−/−^ DN3 cells (**B**) Distribution of p-values versus log2 fold change values for 127 genes identified as being significantly differentially expressed (**C**) Pie-chart showing fraction of 42 genes filtered through stringent criteria (p-value ≤ 5E-05, log2 FC ≥2.0) querying against INTERFEROME database to identify interferon response genes (**D**) INTERFEROME interface was used to categorize IRGs based on their responsiveness to either Type I or Type II interferons. (**E**) qRT-PCR analysis was performed on purified cDNA from DN3 cells differentiated from *tc-ptp* ^+/+^ or *tc-ptp* ^−/−^ LSKs, to quantify the relative abundances of the indicated mRNAs. Each experiment included triplicate technical samples, each normalized to the reference gene *Gapdh*. Data are means+/− SEM of a single experiment and are representative of three independent experiments. Statistical significance was determine by a two-tailed unpaired t-test.

RNA-Seq was used to identify changes to gene programs characteristic of early T cell development. *Tc-ptp* ^+/+^ and *tc-ptp* ^−/−^ DN3 cells were differentiated from LSKs on OP9-DL1 cells. RNA was extracted and subjected to RNA-sequencing. A group of 128 genes was identified as being differentially expressed in *tc-ptp* ^−/−^ DN3 cells as compared to *tc-ptp* ^+/+^ DN3 cells (Fig. 3A, Supplemental Table 1). It has been previously reported that the distinct stages of early T cell development are each associated with gene clusters identifiable by similar expression patterns, each cluster being distinguished by a characteristic gene^25^. No significant overlap was observed between the *tc-ptp* ^−/−^ differentially expressed gene set and gene clusters associated with the loss of progenitor potential, T lineage commitment, Notch signaling or β-selection (Supplemental Fig. 1).

The initial group of 128 genes was then restricted to stringent criteria to include genes with a p-value of 5E-05 or less and a log2 fold difference of at least 2.0 (Fig. 3B, Table 1). The restricted group of 42 genes included multiple members within several gene families partially regulated by the IFN-STAT1 signaling axis, such as the SLFN gene family, the IFN-inducible GTPases and the oligoadenylate synthases (OAS). Such genes have been implicated in the regulation of cell death and cell cycle in response to interferons. When the restricted gene group was queried against the INTERFEROME database^26^, 39 genes were identified as interferon response genes (IRGs), including both type I and type II responses (Fig. 3C,D).

A selection of genes from the restricted 42 gene list were then confirmed by quantitative RT-PCR analysis of purified RNA extracted from *tc-ptp* ^+/+^ and *tc-ptp* ^−/−^ DN3 cells differentiated on OP9-DL1 cells. Genes among the Slfn, GBP and OAS families were confirmed (Fig. 3E).

### The loss of TC-PTP disrupts IL-7R signaling in tc-ptp^−/−^ co-cultures

**Figure 4:**
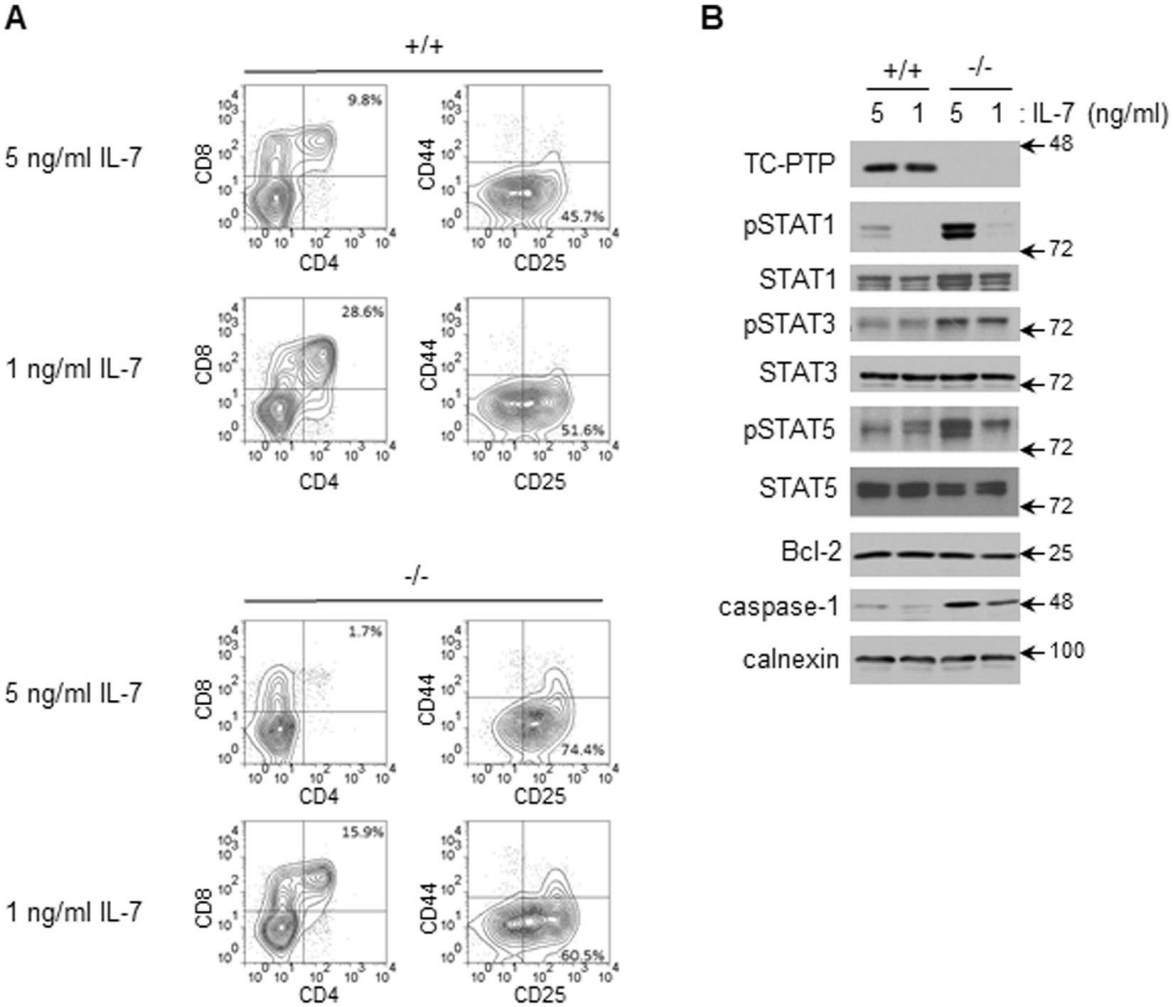
TC-PTP deficiency associated with deregulated activation of STAT family members. (**A**) FACS-sorted *tc-ptp* ^+/+^ and *tc-ptp* ^−/−^ LSKs were differentiated into DN3 cells on OP9-DL1 cells in the presence of 5 ng/ml FLt3L and either 5 ng/ml IL-7 or 1 ng/ml IL-7. The status of progenitor differentiation was assessed by flow cytometry prior to cell harvesting. (**B**) After 21 days of culture, cells were isolated, lysed, and resolved by SDS-PAGE, and proteins detected by western blotting as indicated. Blots are representative of at least 3 experiments. Full-length blots are presented in Supplementary Information.

As described, TC-PTP is also a negative regulator of IFNγ receptor signaling and STAT1 activation. While gene profiling identified an interferon-response gene profile, an upregulation of Type I or Type II interferon gene transcripts were not detected. Given our previous report that the loss of the closely related phosphatase PTP1B results in an alteration in STAT-induced gene profiles, we investigated whether the loss of TC-PTP re-directed IL-7R-mediated STAT gene targeting. Western blotting demonstrated heightened phosphorylation of STAT1, STAT3 and STAT5 in *tc-ptp* ^−/−^ versus *tc-ptp* ^+/+^ co-cultures. Elevated expression of caspase-1, a candidate from the interferon-response gene profile, was also observed (Fig. 4).

The phosphorylation of STAT1, STAT3 and STAT5 titrated with IL-7 concentrations, establishing a direct link between IL-7, alternate STAT activation and changes in gene expression. Expression of the prototypical IL-7-STAT5 gene target *Bcl-2*, did not titrate with recombinant IL-7, suggesting that a level of saturation is reached at low levels of cytokine concentration. (Fig 4).

### Inducible depletion of TC-PTP expression

**Figure 5:**
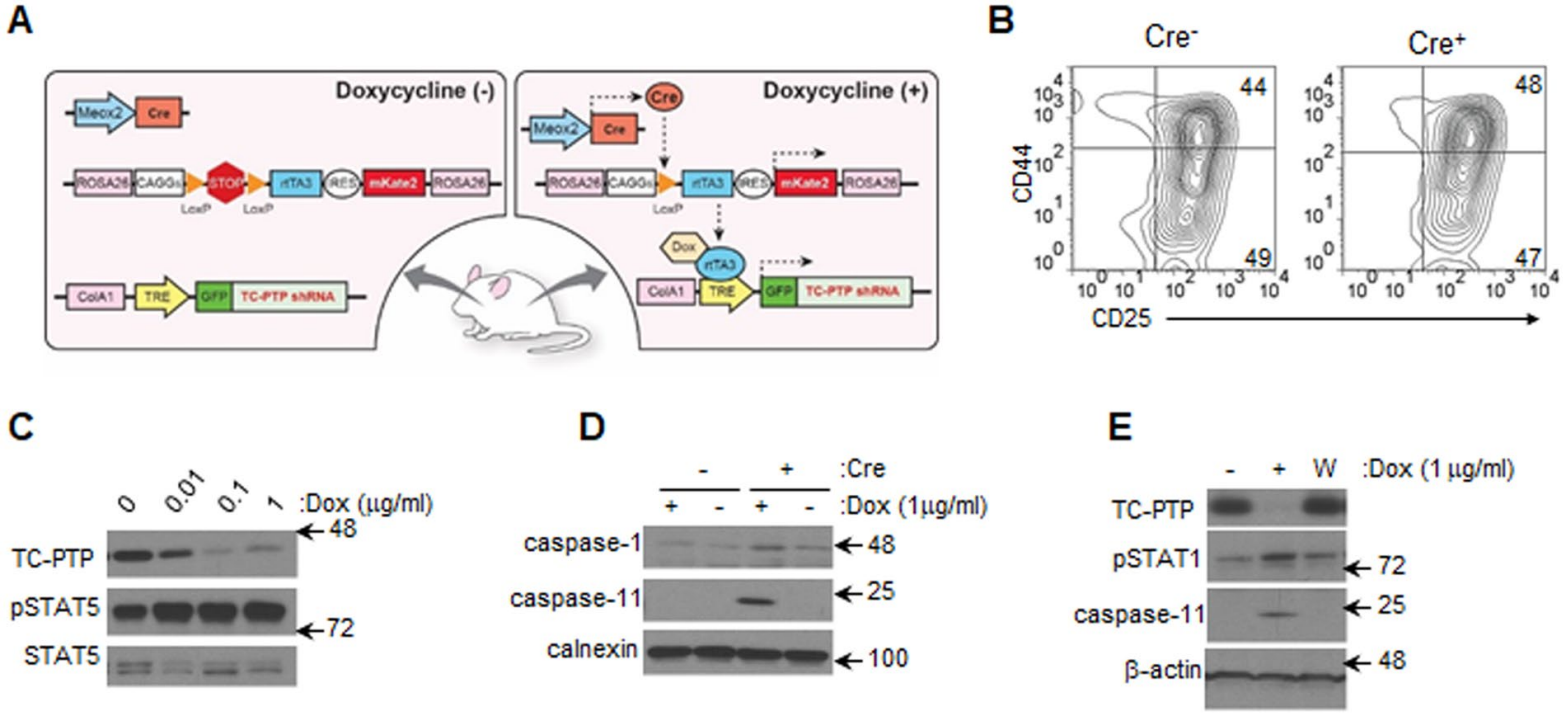
Reversible depletion of TC-PTP permits regulation of transcriptional profile. (**A**) Schematic representation of the genetic components of TSI mouse model. Drawn by Noriko Uetani, Goodman Cancer Research Centre, McGill University. (**B**) LSKs cell sorted from Cre^+^ and Cre^−^ TSI bone marrow and cultured until cultures contained DN2 and DN3 subsets as determine by flow cytometry. (**C**) Titrating doses of doxycycline were added to DN2/DN3 cultures. Following 4 day, progenitors were harvested and cell lysates resolved and probed. (**D**) Cre^+^ and Cre^−^ TSI co-cultures were treated with doxycycline for 4 days after which cell lysates were generated and resolved. (**E**) TC-PTP expression was rescued by washing doxycycline-treated Cre^+^ TSI co-cultures and maintaining co-cultures for 4 more days in the absence of doxycycline (W). Cell lysates were resolved and transferred membranes probed for markers of an interferon-response. Representative blots from 3 independent experiments are shown. Full-length blots are presented in Supplementary Information.

We addressed the possibility that the induction of an interferon response gene profile, resulted from secondary effects linked to a chronic deficiency of TC-PTP. To do so, TC-PTP expression was depleted in an acute and reversible manner using a novel tissue-specific tetracycline-inducible shRNA mouse model (TSI) (Fig. 5A). The TSI mouse is based on two genetic components. First, at the *collagen type I alpha* (Col1A1) locus, a tet response element (TRE) promoter controls expression of GFP. The 3′UTR of the GFP transcript contains a TC-PTP targeting shRNA (*TRE-GFP-shTC-PTP*). Second, at the ROSA 26 locus, a CAGs promoter drives expression of a recombinant reverse tet-transactivator (rtTA) and the far-red fluorescent gene *mKATE2* downstream of an internal ribosomal entry site (IRES). To allow for conditional gene expression, a LoxP-flanked polyadenyation signal (*LSL*) was cloned upstream of the rtTA (*CAGs-LSL-rtTA3-IRES-mKate2*)^27^. To induce rtTA expression, the *LSL* cassette is excised by crossing TSI mice (homozygous for both *TRE-GFP-shTCPTP* and *CAGs-LSL-rtTA3-IRES-mKate2)* with a tissue-specific Cre recombinase mouse strain. In all experiments presented here, TSI mice were crossed with *Meox2-Cre* mice, causing global expression of the rtTA. Expression of GFP and the TC-PTP shRNA however, are only induced upon administration of doxyxycline (Dox, tetracycline analog) which enables rtTA to bind to the TRE promoter (Fig. 5A).

The inducible nature of the mouse model was tested *in vivo* using experimental TSI/Cre^+^ mice and control TSI/Cre^−^ mice. Mice were treated 7 days with previously reported optimal concentration of 1mg/ml of Dox and *in vivo* GFP monitoring demonstrated the induction of the system (Suppl. Figure 2A). Further analysis of the inducible property of the system was performed by giving mice titrating doses of Dox for 7 days after which they were sacrificed and thymii were harvested. The expression of GFP in thymii was monitored by fluorescence microscopy as well as FACS (Suppl. Figure 2B). Gradually increased levels of GFP expression can be visualized by microscopy and flow cytometry in the thymus of the TSI/cre+ mice, but not in the control TSI/cre- mice. A parallel between the exponential expression of GFP and the KD of TC-PTP could also be observed by Western blots performed on the thymic lysates isolated from the corresponding mice (Suppl. Figure 2C). The *in vivo* system generated allowed for a tight regulation of the shRNA expression given that neither GFP expression nor KD of TC-PTP were observed in the control TSI/cre- mice treated with Dox.

### Rescuing TC-PTP expression suppresses pro-inflammatory gene set

LSKs isolated from the bone marrow of Cre^-^ and Cre^+^ TSI mice and differentiated for 12 days on OP9-DL1 cells displayed similar CD44/CD25 profiles (Fig. 5B). The addition of titrating doses of Dox to Cre^+^ TSI cultures for 4 days caused decreasing levels of TC-PTP expression corresponding with increasing STAT5 phosphorylation (Fig. 5C). Depletion of TC-PTP expression resulted in the induction of caspase-1 and caspase-11 expression, both candidates from the interferon-response gene profile, which was not observed in Cre^-^ TSI cultures. (Fig 5D). Subsequent removal of Dox rescued TC-PTP expression, decreased STAT phosphorylation and both caspase 1 and caspase 11 expression (Fig. 5E). As such, we can confirm that the induction of an interferon gene program in TC-PTP depleted progenitors is reversible and therefore not a reflection of a fundamental change in the programming or differentiation of the cells.

## Discussion

The current study identifies an additional regulatory mechanism controlling JAK-STAT activation during T cell differentiation. The loss of TC-PTP expression in early T cell progenitors is shown to increase the phosphorylation of multiple STAT family members, induce an IFN-response gene profile, and perturb T cell development.

Multiple regulatory mechanisms determine the specificity of JAK-STAT signaling in response to CytR activation. While most CytRs activate a dominant STAT family member, in its absence an alternate STAT can be used although the transcriptional output and the nature of the elicited gene program changes^28^. Alternatively, STAT family members may work co-operatively to drive transcriptional output and specificity. Such is the case for IL-6 and IL-27, which both activate STAT1 and STAT3. While STAT3 is the primary driver of transcriptional output, STAT1 provides CytR specificity^29^. The specificity of gene programs elicited by CytR is therefore in part regulated by the availability and ratio of activated STAT family members within a cell at a given time.

In the case of the IL-7, STAT5 is the primary STAT family member activated downstream of the receptor^30^. As observed with the majority of common γ chain CytRs however, the IL-7R does have the capacity to activate STAT1, albeit at levels insufficient to provoke an IFN-transcriptional response. We have proven here that TC-PTP is critical in suppressing robust STAT1 activation following IL-7 stimulation, and is therefore required to maintain the necessary ratio of activated STAT family members and ensure the specificity of the IL-7R response.

In addition to the regulation of specificity, molecular mechanisms also control the amplitude and duration of STAT activation. These include the internalization and degradation of cytokine receptors by the lysosome or proteasome pathways, the recruitment of cytokine-induced SH2-containing protein 1 (CIS1) and suppressors of cytokine signaling proteins (SOCS 1–7), and the targeting of STAT molecules for SUMoylation by the protein inhibitors of STAT (PIAS) proteins. These mechanisms however influence the amplitude of the response rather than the nature of the response. In comparison, TC-PTP can modulate both the extent of STAT phosphorylation, and influence gene targeting by maintaining the activation of a dominant STAT family member.

T cells in the later stage of development, specifically those beyond positive selection, reside within the thymic medulla and are in contact with interferons. At this developmental stage however, low grade tonic IFN-STAT1 signaling is required for the final stages of maturation prior to emigration from the thymus. The tonic signals were proposed to prime T cells for the capacity to respond to inflammatory cytokines^31^. We would suggest that the loss of TC-PTP in mature thymocytes augments the low grade IFN-STAT1 signaling which, when combined with the aberrant IL-7-STAT1 axis, has detrimental effects on T cell differentiation.

Under healthy conditions, we have proven that TC-PTP is not solely required to balance the activity of the JAK family members. Rather our findings have demonstrated that TC-PTP’s role in supporting T cell development is related to its role in tailoring STAT gene targeting and as a consequence modifying cellular behavior to drive T cell differentiation.

Varying patterns of chronic STAT1, STAT3 and STAT5 activation have been identified in both B and T cell chronic and acute leukemias^32^. While the loss of TC-PTP has been associated with increased STAT activation in myeloid and lymphoid cell lineages, biallelic inactivation mutations in the *PTPN2* gene have only been identified in the TLX^+^ sub-group of T-ALL, and not in B cell leukemia and lymphoma^33–35^.

The mechanism by which TC-PTP loss contributes to T cell transformation was attributed to elevated STAT activation given that TC-PTP deficient primary leukemic T cells gain a proliferative advantage in response to IL-2 and IL-7. However, the oncogene NUP214-ABL1 has been identified as a TC-PTP substrate, and TC-PTP depletion renders lymphoid cells more susceptible to NUP214-ABL1 mediated transformation^33^. Such evidence, suggests that the loss of TC-PTP may contribute to T cell leukemia by modulating multiple pathways, driving complex alterations in gene expression. It is therefore of interest to determine the molecular interplay between TLX ectopic expression, expression of NUP214-ABL1 and TC-PTP depletion. In particular, it will be worthwhile to determine how aberrant gene expression due to elevated STAT1, 3 and 5 activity is involved.

## Electronic supplementary material

Supplementary Information
