# Supplementary material for: TC-PTP regulates the IL-7 transcriptional response during murine early T cell development
Source: Sci Rep. 2017 Oct 16;7:13275. doi: 10.1038/s41598-017-13673-w (PMC5643372; doi:10.1038/s41598-017-13673-w)
Supplement: Supplementary file 1 — Supplementary Information [file 41598_2017_13673_MOESM1_ESM.pdf]

TC-PTP REGULATES THE IL-7 TRANSCRIPTIONAL RESPONSE DURING MURINE EARLY T CELL DEVELOPMENT

K.A. Pike[1], T. Hatzihristidis[1,2], S. Bussières-Marmen[1,3], F. Robert[1], N. Desai[1,3], D. Miranda-Saavedra[4,5], J. Pelletier[1,2] and M.L. Tremblay[1,2,3]

[1]Rosalind and Morris Goodman Cancer Centre, McGill University, Montréal QC H3A 1A3, Canada.[2]Division of Experimental Medicine, Department of Medicine, McGill University, Montréal, QC H3A 1A3, Canada. [3]Department of Biochemistry, McGill University, Montréal QC H3A 1A3, Canada.[4]Centro de Biología Molecular Severo Ochoa, CSIC/Universidad Autónoma de Madrid, 28049 Madrid, Spain. [5] Department of Computer Science, University of Oxford, Wolfson Building Parks Road, OXFORD, OX1 3QD, UK

Corresponding author:
M.L. Tremblay
1160 Pine Avenue West
Office Room 612; Lab Room 603
Montreal, Quebec H3A 1A3
Tel (514) 398-7290
Fax (514) 398-6769
Email: michel.tremblay@mcgill.ca

Short title: TC-PTP, IL-7 and T cell development

Supplementary Information

Supplemental Table S1. Differentially expressed genes in tc-ptp-/- DN3 cells differentiated in OP9-DL1 co-culture compared to tc-ptp+/+ DN3 cells. Average fragments per kilobase (FPKM) derived from 3 biological replicates.

ENSEMBL ID	Gene Symbol	WT (AVG FPKM)	KO (AVG FPKM)	log2 (fold_change)	p_value
ENSMUST00000183203	Gm26969	0	2.86973	inf	5.00E-05
ENSMUST00000061185	Krt81	0	0.907917	inf	5.00E-05
ENSMUST00000090391	Pax6	0.00641769	0.752857	6.87418	5.00E-05
ENSMUST00000037994	Slfn1	0.629762	49.7789	6.30458	5.00E-05
ENSMUST00000113879	H2-Q8	3.56484	272.398	6.25574	5.00E-05
ENSMUST00000032737	Trpm1	0.0878201	6.46333	6.20158	5.00E-05
ENSMUST00000030396	Ermap	0.023009	1.08066	5.55356	0.0001
ENSMUST00000086451	Nos1	0.0628811	2.93379	5.544	0.0003
ENSMUST00000071951	H2-Q7	20.7694	614.774	4.88752	5.00E-05
ENSMUST00000046739	Ifi44l	1.7456	50.7077	4.86041	0.0002
ENSMUST00000163239	Spats2l	0.0778114	2.1568	4.79276	5.00E-05
ENSMUST00000087983	Il18r1	0.157793	4.2696	4.758	0.00045
ENSMUST00000026912	1700008F21Rik	0.0806828	2.16035	4.74286	0.00025
ENSMUST00000135184	Mx2	0.228171	5.05184	4.46862	0.0001
ENSMUST00000023655	Mx2	1.47454	31.8572	4.43328	5.00E-05
ENSMUST00000116010	H2-Q5	4.70419	70.6167	3.90799	5.00E-05
ENSMUST00000082687	AF357355	0.714356	10.234	3.84059	5.00E-05
ENSMUST00000029671	Ifi44	6.74574	93.2798	3.78952	5.00E-05
ENSMUST00000015581	Gzmb	3.38683	46.1458	3.76819	5.00E-05
ENSMUST00000112467	Gm14446	9.47289	126.534	3.73958	5.00E-05
ENSMUST00000076249	I830012O16Rik	2.17103	28.969	3.73805	5.00E-05
ENSMUST00000023248	Ly6a	64.8023	748.919	3.53069	5.00E-05
ENSMUST00000102825	Ifit3	12.7917	147.588	3.52829	5.00E-05
ENSMUST00000037976	Pydc4	3.11267	34.6213	3.47543	5.00E-05
ENSMUST00000031549	Gm4951	15.6324	166.136	3.40975	5.00E-05
ENSMUST00000065408	Ly6c1	23.0249	230.51	3.32356	5.00E-05
ENSMUST00000029936	Gbp2b; LOC102643136; Gbp5	6.08113	57.5039	3.24125	5.00E-05
ENSMUST00000090406	BC094916	2.4224	22.262	3.20008	0.0003
ENSMUST00000020970	Rsad2	2.15876	18.7161	3.11601	5.00E-05
ENSMUST00000053909	Oas2	8.88515	75.7074	3.09097	5.00E-05
ENSMUST00000102642	Ube2l6	6.86829	55.3052	3.00939	5.00E-05
ENSMUST00000046838	Adam22	0.0714853	0.557776	2.96397	0.0001
ENSMUST00000067443	Slfn5	9.76887	74.2155	2.92546	5.00E-05
ENSMUST00000108825	Gm12253	1.95832	13.2333	2.75649	5.00E-05
ENSMUST00000029018	Zbp1	29.7782	193.714	2.7016	5.00E-05
ENSMUST00000044833	Oas3	9.68588	60.4577	2.64197	5.00E-05
ENSMUST00000057725	Samhd1	9.7597	60.879	2.64104	5.00E-05
ENSMUST00000165774	Gbp2	14.2193	87.9553	2.62892	5.00E-05
ENSMUST00000103682	Trdj1	326.554	1968.53	2.59173	0.0002
ENSMUST00000015667	Ctss	5.46336	32.7083	2.5818	5.00E-05
ENSMUST00000119467	Gm12250	21.965	129.957	2.56475	5.00E-05
ENSMUST00000080322	Oas1a	13.9428	81.2341	2.54256	5.00E-05
ENSMUST00000026564	Ifitm1	45.5963	258.185	2.50142	5.00E-05
ENSMUST00000053085	Nlrc5	14.4204	79.3806	2.46068	5.00E-05
ENSMUST00000035938	Ccl5	11.9155	65.3638	2.45565	0.00035

ENSMUST00000027015	Casp1	9.67395	52.0372	2.42737	5.00E-05
ENSMUST00000055071	Ifi27l2a	106.989	557.24	2.38084	5.00E-05
ENSMUST00000102824	Ifit1	15.4928	78.9642	2.3496	5.00E-05
ENSMUST00000090127	Gbp5; Gbp2b; LOC102643136	6.25477	31.7183	2.34229	5.00E-05
ENSMUST00000032198	Usp18	30.253	149.024	2.3004	5.00E-05
ENSMUST00000035266	Igtp	59.4418	289.992	2.28646	5.00E-05
ENSMUST00000093902	Rnf213	35.776	171.866	2.26422	5.00E-05
ENSMUST00000166121	Phf11b	7.87794	37.4118	2.2476	0.0001
ENSMUST00000020969	Cmpk2	5.59195	25.8325	2.20777	5.00E-05
ENSMUST00000035258	Ms4a4b	64.4905	280.406	2.12036	5.00E-05
ENSMUST00000070968	Stat1	60.509	260.381	2.1054	5.00E-05
ENSMUST00000040750	Lif	21.5133	62.1575	2.09417	5.00E-05
ENSMUST00000027012	Casp4	8.1896	34.8142	2.08781	5.00E-05
ENSMUST00000068291	H2-Q10	2.67868	10.8627	2.01979	0.0001
ENSMUST00000078778	Gp49a	4.59448	18.5145	2.01068	0.0001
ENSMUST00000085876	Pydc3	12.6929	50.9631	2.00544	5.00E-05
ENSMUST00000056071	Pyhin1	4.68637	18.2663	1.96265	5.00E-05
ENSMUST00000031817	Herc6	13.0898	50.8652	1.95824	5.00E-05
ENSMUST00000094041	Xaf1	65.4447	254.01	1.95654	5.00E-05
ENSMUST00000074570	Gpr114	7.13737	27.5439	1.94827	0.00025
ENSMUST00000025181	H2-K1	338.627	1288.58	1.92801	5.00E-05
ENSMUST00000099676	AW112010	19.8269	74.2993	1.90589	0.00015
ENSMUST00000028259	Ifih1	15.0541	56.2696	1.9022	5.00E-05
ENSMUST00000085425	Isg15	130.44	486.806	1.89996	5.00E-05
ENSMUST00000024112	Mx2	13.9259	48.1023	1.78833	5.00E-05
ENSMUST00000029935	Gbp3	16.5918	57.0892	1.78275	5.00E-05
ENSMUST00000042665	Parp14	18.7993	64.3647	1.77559	5.00E-05
ENSMUST00000096358	Apol7e	4.68588	15.9123	1.76375	0.00015
ENSMUST00000086377	Oas1b	16.4042	54.256	1.72571	5.00E-05
ENSMUST00000042312	Trafd1	31.1679	102.614	1.7191	5.00E-05
ENSMUST00000049519	Irgm1	60.0635	196.841	1.71247	5.00E-05
ENSMUST00000081435	H2-Q4	118.076	385.926	1.70861	5.00E-05
ENSMUST00000172503	H2-L; H2-D1	265.192	847.494	1.67616	5.00E-05
ENSMUST00000025161	Tapbp, Zbtb22	73.2221	233.813	1.67501	5.00E-05
ENSMUST00000166912	Phf11c	8.77498	27.733	1.66014	5.00E-05
ENSMUST00000038398	Parp12	17.2848	54.1481	1.6474	5.00E-05
ENSMUST00000079625	Tor3a	6.27696	19.5228	1.63703	0.00015
ENSMUST00000000266	LOC100044068; Ifi202b	16.3232	49.019	1.58641	5.00E-05
ENSMUST00000026565	Ifitm3	118.867	356.251	1.58354	5.00E-05
ENSMUST00000051672	Bst2	195.308	581.922	1.57507	5.00E-05
ENSMUST00000120087	Samd9l	36.6908	107.086	1.54528	5.00E-05
ENSMUST00000102826	Ifit2	14.4707	41.9052	1.53399	5.00E-05
ENSMUST00000076922	Trim30a	52.5106	151.011	1.52397	0.0001
ENSMUST00000079421	Daxx	41.8491	119.439	1.513	5.00E-05
ENSMUST00000026737	Shisa5	266.256	752.039	1.49799	0.00015
ENSMUST00000045097	Gbp7	36.3936	97.9911	1.42897	5.00E-05
ENSMUST00000038141	Slfn8	20.7402	55.7521	1.4266	5.00E-05

ENSMUST00000037907	Ddx58	34.7581	91.6265	1.39842	0.0001
ENSMUST00000046383	Tnfsf10	6.50432	17.0658	1.39164	5.00E-05
ENSMUST00000085673	Pml	44.3396	115.177	1.37718	0.0002
ENSMUST00000038099	Socs1	41.1144	104.955	1.35205	5.00E-05
ENSMUST00000094203	Helz2	24.7235	62.8659	1.34639	5.00E-05
ENSMUST00000011400	Adam19	12.2257	29.8045	1.28561	0.00015
ENSMUST00000027153	Acadl	70.5354	171.53	1.28204	5.00E-05
ENSMUST00000102476	B2m	1287.77	3130.89	1.2817	5.00E-05
ENSMUST00000067506	Gimap4	91.5138	220.638	1.26962	5.00E-05
ENSMUST00000038142	Aen	73.541	175.164	1.25208	0.0003
ENSMUST00000049460	Grn	19.4665	46.0455	1.24206	0.00025
ENSMUST00000001327	Itgb7	21.7548	51.2359	1.23583	5.00E-05
ENSMUST00000006101	Itgae	31.1656	72.6334	1.22068	0.0004
ENSMUST00000085708	Stat2	15.5566	36.1931	1.21819	5.00E-05
ENSMUST00000168747	Atp10a	7.62951	17.6194	1.2075	0.0001
ENSMUST00000040128	Atp8b4	70.9598	161.123	1.18309	5.00E-05
ENSMUST00000043722	Lgals3bp	120.738	271.002	1.16642	0.0001
ENSMUST00000038423	Rtp4	62.6863	139.944	1.15862	5.00E-05
ENSMUST00000010502	Ifi35	37.4774	83.2698	1.15177	0.0004
ENSMUST00000033264	Trim21	12.8072	27.9262	1.12466	0.0002
ENSMUST00000025262	Ltb	94.7336	202.385	1.09515	5.00E-05
ENSMUST00000021028	Itgb3	11.1757	23.8459	1.09338	0.0001
ENSMUST00000089398	Il2rb	21.0104	43.7536	1.0583	0.00015
ENSMUST00000031264	Plac8	905.944	1863.27	1.04035	0.0001
ENSMUST00000021632	Akr1c12	147.2	74.6417	-0.979723	0.00045
ENSMUST00000099945	Amd1	19.9629	9.0657	-1.13883	0.0004
ENSMUST00000088040	Pcna-ps2	91.0827	39.7508	-1.19619	0.0002
ENSMUST00000032309	Ybx3	45.5864	18.2155	-1.32343	0.0001
ENSMUST00000024894	Cyp1b1	13.2277	3.63225	-1.86463	5.00E-05
ENSMUST00000001147	Col6a1	15.9353	4.37531	-1.86477	0.00015
ENSMUST00000001181	Col6a2	11.7242	3.08309	-1.92704	5.00E-05
ENSMUST00000033741	Bgn	48.5775	11.8487	-2.03556	0.00015
ENSMUST00000055226	Fn1	19.2389	4.51639	-2.09078	5.00E-05
ENSMUST00000073043	Cxcl12	52.6659	11.4868	-2.19689	5.00E-05
ENSMUST00000025409	Lox	12.9108	2.8119	-2.19896	5.00E-05
ENSMUST00000039631	Acta2	18.2207	3.76885	-2.27338	0.00025

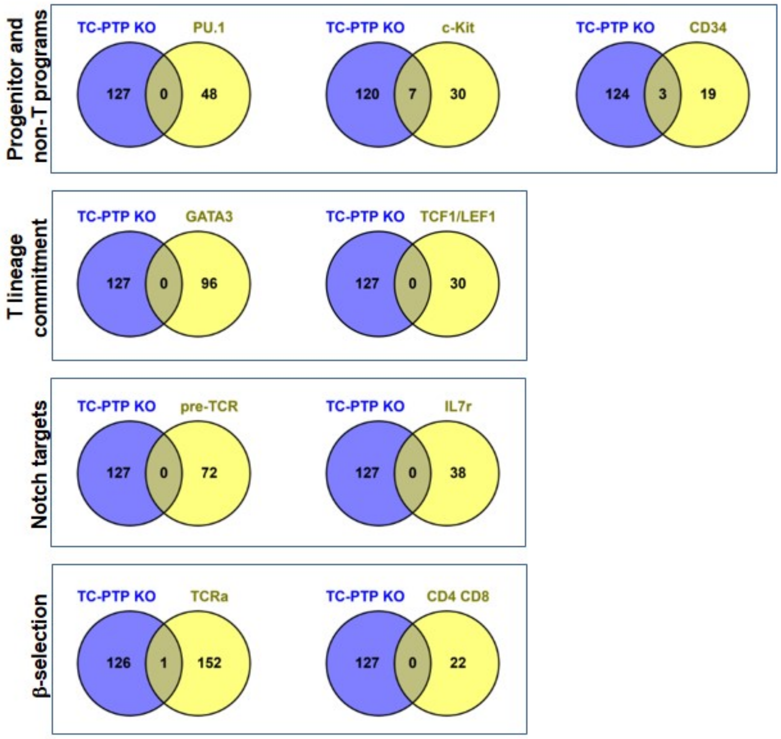

Supplemental Figure S1. It has been previously reported that the distinct stages of early T cell development are each associated with gene clusters identified by similar patterns of expression. Each cluster being identified by a characteristic gene[27]. No significant overlap was observed between the tc-ptp-/- differentially expressed gene set and gene clusters associated with the loss of progenitor potential, T lineage commitment, Notch signaling or β-selection.

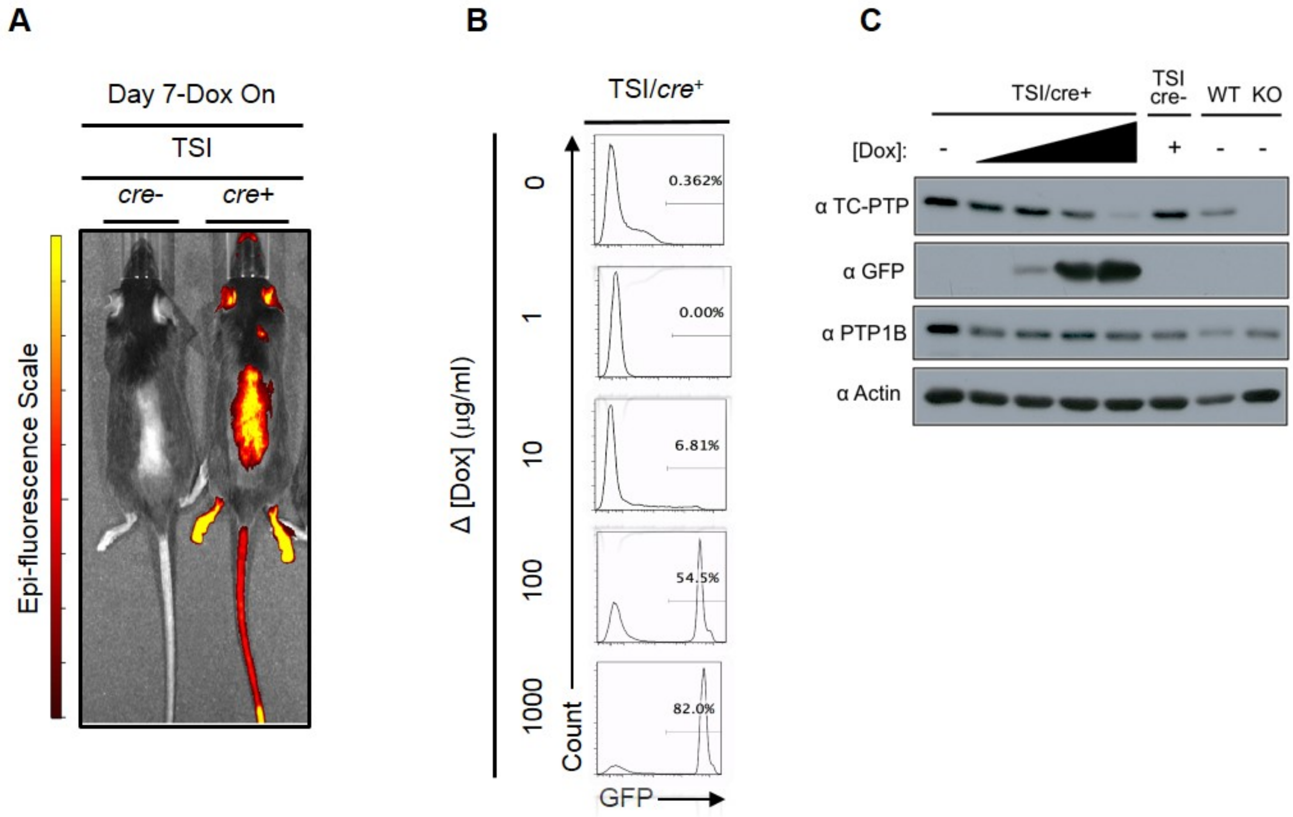

Supplemental Figure S2. Generation of an inducible TC-PTP shRNA mouse. (A) In vivo monitoring of GFP expression in mice treated 7 days with Dox (1mg/ml). Epi-fluorescence scale in photons/second (bar = 0.5x10-3) where bright yellow to darker red correlates with high to low expression of GFP. D) Western blot on thymic cells isolated from TSI/cre, mice, TSI/cre+ mice with the shRNA only on one allele (sh/WT) or both alleles of ColA1 (sh/sh). All mice were treated with Dox (1mg/ml) for 7 days. (B) Thymic cells from the TSI/cre+ mouse treated with different concentrations of Dox (0-1000 ug/ml). Percentage of GFP+ cells assessed by flow cytometry, gated on live cells (7AAD-). (C) Protein analysis of thymii from mice receiving the water treatment with different concentrations of Dox ranging from 0,1,10,100,1000 ug/ml (+: 1000ug/ml). Membrane blotted for TC-PTP, GFP, p-STAT1, STAT1, PTP-1B and actin as a loading control.

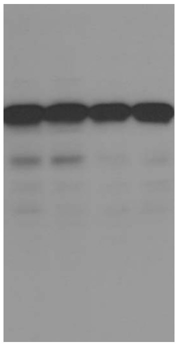

Figure 1B - Actin

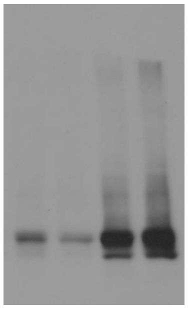

Figure 1B - pSTAT5

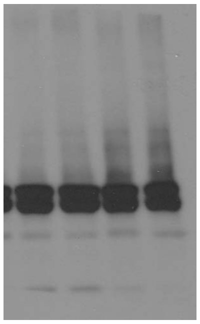

Figure 1B - STAT5

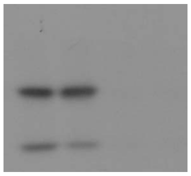

Figure 1B - TCPTP

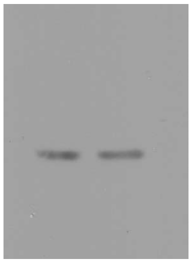

Figure 2B - Actin

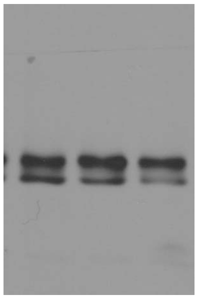

Figure 2B - STAT5

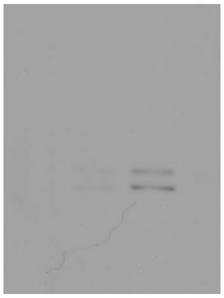

Figure 2B - pSTAT5

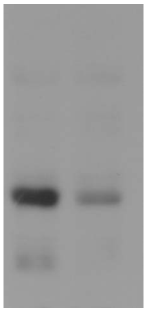

Figure 2B - TCPTP

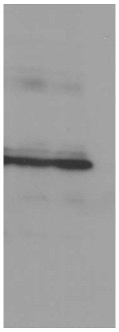

Figure 2D - Actin

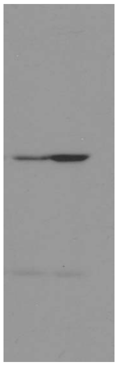

Figure 2D - pSTAT5

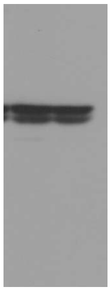

Figure 2D - STAT5

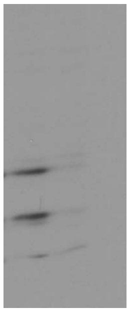

Figure 2D - TCPTP

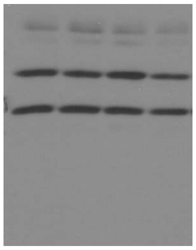

Figure 4 - Bcl2

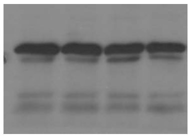

Figure 4 - Calnexin

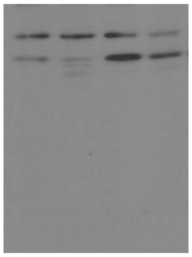

Figure 4 - Caspase1

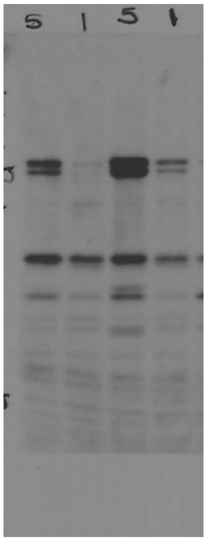

Figure 4 - pSTAT1

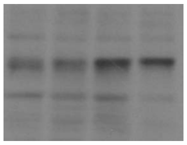

Figure 4 - pSTAT3

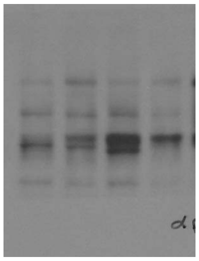

Figure 4 - pSTAT5

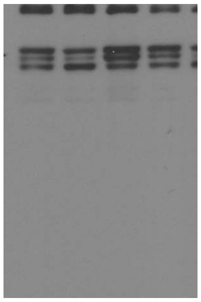

Figure 4 - STAT1

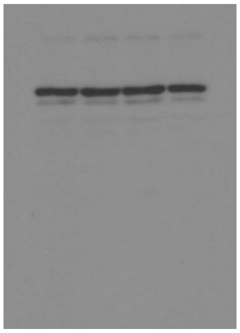

Figure 4 - STAT3

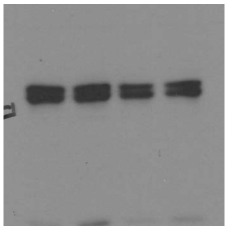

Figure 4 - STAT5

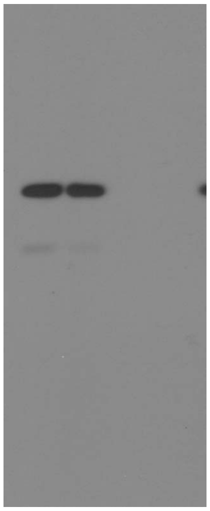

Figure 4 - TCPTP

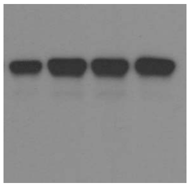

Figure 5C - pSTAT5

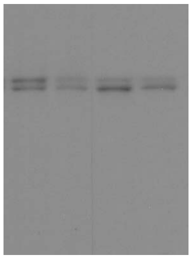

Figure 5C - STAT5

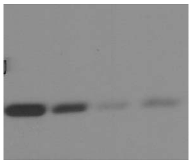

Figure 5C - TCPTP

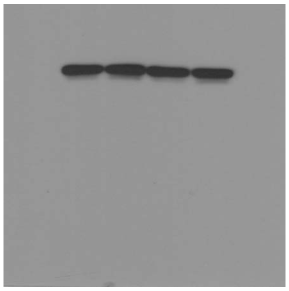

Figure 5D - Calnexin

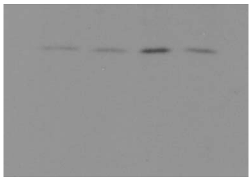

Figure 5D - Caspase1

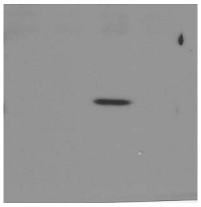

Figure 5D - Caspase11

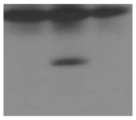

Figure 5E - Caspase11

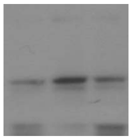

Figure 5E - pSTAT1

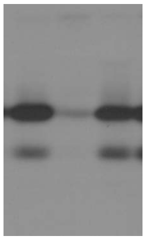

Figure 5E - TCPTP
